# Mutations in Putative Mutator Genes of *Mycobacterium tuberculosis* Strains of the W-Beijing Family

**DOI:** 10.3201/eid0907.020803

**Published:** 2003-07

**Authors:** Mina Ebrahimi Rad, Pablo Bifani, Carlos Martin, Kristin Kremer, Sofia Samper, Jean Rauzier, Barry Kreiswirth, Jesus Blazquez, Marc Jouan, Dick van Soolingen, Brigitte Gicquel

**Affiliations:** * Institut Pasteur, Paris, France; † Institut Pasteur, Lille, France; ‡ Universidad de Zaragoza, Zaragoza, Spain; § National Institute of Public Health and the Environment, Bilthoven, the Netherlands; ¶ Public Health Research Institute, Newark, New Jersey, USA; # National Institute of Health, Madrid, Spain

**Keywords:** tuberculosis, epidemic, antibiotic resistance, mutator, research

## Abstract

Alterations in genes involved in the repair of DNA mutations (*mut* genes) result in an increased mutation frequency and better adaptability of the bacterium to stressful conditions. W-Beijing genotype strains displayed unique missense alterations in three putative *mut* genes, including two of the *mutT* type (Rv3908 and *mutT2*) and *ogt*. These polymorphisms were found to be characteristic and unique to W-Beijing phylogenetic lineage. Analysis of the *mut* genes in 55 representative W-Beijing isolates suggests a sequential acquisition of the mutations, elucidating a plausible pathway of the molecular evolution of this clonal family. The acquisition of *mut* genes may explain in part the ability of the isolates of W-Beijing type to rapidly adapt to their environment.

Tuberculosis (TB) and AIDS cause more deaths in adults worldwide than any other infectious disease. Globally, the number of TB cases is growing at a rate of 2% per year. Resistance, especially multidrug-resistance (MDR), is an increasing problem (*1*) and a growing hazard to human health. Many outbreaks of MDR-TB, defined as resistance to at least rifampicin and isoniazid, have been reported, with poor response to therapy and very high disease and death rates. Some TB outbreaks have involved patients with HIV co-infection (*2*,*3*). Although in several instances, MDR outbreaks associated with a particular genotype, such as the W strain, have been identified (*4*,*5*), drug-susceptible variants of the W strain account for most of this group of isolates characterized to date.

In 1995, the largest proportion of the *Mycobacterium tuberculosis* strains from Beijing, China, shared a high degree of similarity in IS*6110* restriction fragment length polymorphism (RFLP) patterns and identical spoligo patterns (*6*). Subsequent molecular analyses have indicated that the W and Beijing isolates constitute a single group of strains designated as the W-Beijing genotype (Figure 1). The global distribution and success of *M. tuberculosis* isolates of the W-Beijing genotype have led to the hypothesis that these strains may have selective advantages over other *M. tuberculosis* strains. In addition to the W-MDR strain identified in New York City, and areas in Cuba, Estonia, Vietnam, and Russia, the W-Beijing genotype has been significantly associated with drug resistance (7 and unpub. data). Several studies have suggested that the W-Beijing genotype strains are disseminating throughout the world (*7*). In Vietnam, the proportion of W-Beijing strains was 71% in patients <25 years of age and 41% for those >25 years of age (*8*). Furthermore, W-Beijing strains have been implicated in several TB epidemics globally, including ones in New York, Texas, California, South Carolina, and New Jersey in the United States (*9*) and South Africa, Russia, and Spain (*10*). A recent study showed that 82% of MDR strains isolated in a prison in Azerbaijan, Eastern Europe, are of the W-Beijing genotype (*11*).

**Figure 1:**
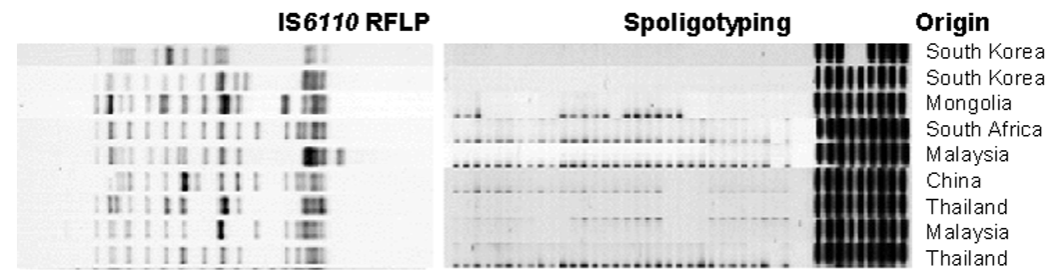
Characteristic patterns of *Mycobacterium tuberculosis* Beijing genotype strains.

Ongoing research is focused on identifying the factors responsible for the worldwide spread of the W-Beijing strains and their ability to adapt and enhance their pathogenicity or virulence. Identifying a possible mechanism for increased adaptation of these bacteria to the human immunologic host defense system or human interventions such as anti-TB treatment is of the utmost importance. Such mechanisms may indicate how the bacterium adapts to the host, a prerequisite for an enhanced accumulation of genomic mutations associated with resistance. In *M. tuberculosis,* resistance to antibiotics occurs because of genomic mutations in certain genes, such as the *katG* gene for isoniazid (INH) resistance and the *rpoB* gene for rifampicin resistance (*12*). In contrast to several other pathogens with MDR phenotypes, plasmid or transposon-mediated mechanisms of resistance have not been reported in *M. tuberculosis* (*13*–*15*). Since resistance to bacteriostatic in *M. tuberculosis* is exclusively due to genomic mutations, the bacterium would benefit from an increased mutation rate.

Recent studies provided evidence for a role of mutator phenotypes in the emergence of MDR clinical *Pseudomonas* isolates (*16*). Such phenotypes not only enable the bacteria to acquire resistance to antibiotics more easily but also facilitate their adaptation to a new niche. Bacteria can escape immune surveillance by modulating bacterial resistance to host defense mechanisms (*16*–*18*). This finding prompted us to investigate whether a similar situation exists in *M. tuberculosis*. We have undertaken a comprehensive comparative sequence analysis of selected target genes to evaluate and study the presence of mutations in putative genes expected to play a role in the mutation frequency in such strains.

Mutated phenotypes commonly result from defects in DNA repair (*19*). An in silico analysis suggested that most mismatch repair systems (e.g., *mutS, mutL,* or *mutH*) were missing in the *M. tuberculosis* genome (*20*). However, the frequency of spontaneous mutations in *M. tuberculosis* (in vitro cultures) is similar to that found in other bacteria-carrying mismatch repair systems (*21*), which suggests that other DNA repair mechanisms must be present. Hypothetical open reading frames (ORF), similar to genes known to be responsible for the avoidance or repair of DNA lesions resulting from the alkylation or oxidation of nucleotides, are present in the genome of *M. tuberculosis*. We searched for variations in these genes in 139 clinical isolates to detect possible mutations that could allow an enhanced adaptability to the host and increased resistance to anti-TB drugs.

## Methods

We searched for *mut* genes variation in 139 *M. tuberculosis* complex strains originating from 35 different countries. Ninety-four of these strains were selected because they were representative strains characterized with 13 different genetic markers in previous studies (*6*,*22*).

This set comprised 125 *M. tuberculosis* strains, 1 *M. africanum*, 8 *M. bovis*, 3 *M. bovis* BCG, and 2 *M. microti*. Fifty-five strains had a W-Beijing genotype; 12 had an MDR phenotype. Strains representing different branches of the W-Beijing genotype were studied. Eight MDR *M. tuberculosis* strains with a genotype other than Beijing were included. Five *M. tuberculosis* strains of the W-Beijing genotype and three strains of unrelated genotype were obtained from the national program for surveillance of MDR tuberculosis in Spain. Four *M. tuberculosis* W-Beijing genotype strains isolated in the Netherlands and one from Vietnam were included because they showed spoligo patterns with fewer than nine spacers. Five other W-Beijing genotype strains showed hybridization to an additional spacer, as demonstrated by using the extended set of spacers, two of which lacked hybridization to spacer 37. Strain W4 is part of a drug-susceptible outbreak in New Jersey (*4*); W147 is a drug-resistant isolate widely spread in Russia (*23*). Eleven strains were representative of ancestral W-Beijing strains, which diverged early in the evolution of the W-Beijing phylogenetic lineage. Finally, 29 strains of another frequently observed genotype, the Haarlem genotype (*6*), were investigated.

The collection consisted of 55 W-Beijing genotype isolates, 29 Haarlem genotype isolates, 8 strains of the African genotype, 1 *M. bovis* strain, and 46 representatives of other genotypes. Principal genetic grouping (PGG), according to the polymorphism in *katG* and *gyrA*, was known (*24*) for most of the isolates in this study. Seventy-four strains belong to PGG 1, 54 to PGG 2, and 3 to PGG 3. All isolates were subjected to at least IS*6110* RFLP typing and spoligotyping (*6*). Drug susceptibility was determined for 41 of 139 strains (Table 1 and 2). Several putative *mut* genes were annotated as such in the released genome sequence of *M. tuberculosis* (*25*). In addition, using the BLAST program (*26*), we identified Rv3908 as an ORF carrying a *mutT* domain (*27*) and have since named it *mutT4*.

**Table 1:** Characteristics of *Mycobacterium tuberculosis* complex strains originating from 35 different countries^a^

Strains	Genotype	No. isolates	Country of isolation	Group	*mutT2*	*mutT4*	ogt
ZA20/65	W-Beijing	2	Spain	1	wt	Arg CGG 48 GGG Gly	Arg CGC 37 Leu CTC
ZA67-69	W-Beijing	3	Spain	1	Gly GGA 58 CGA Arg	Arg CGG 48 GGG Gly	Gly GGG 12 Gly GGA
ZA11/16	Haarlem	2	Spain	2	wt	wt	Thr ACC 15 Ser AGC
ZA12-14/17	other	4	Spain	nd	wt	wt	wt
ZA19	*M. bovis*	1	Spain		wt	wt	wt
ZA15	other	1	Spain	nd	wt	wt	wt
ZA60-62	W-Beijing	3	Spain	1	wt	Arg CGG 48 GGG Gly	Arg CGC 37 Leu CTC
CDC1551		1	USA	2	wt	wt	wt
H37Rv		1	USA	3	wt	wt	wt
MT210	W-Beijing	1	USA	1	Gly GGA 58 CGA Arg	Arg CGG 48 GGG Gly	Gly GGG 12 Gly GGA
20	W-Beijing	1	Mongolia	1	Gly GGA 58 CGA Arg	Arg CGG 48 GGG Gly	Gly GGG 12 Gly GGA
30	W-Beijing	1	South Africa	1	Gly GGA 58 CGA Arg	Arg CGG 48 GGG Gly	Gly GGG 12 Gly GGA
34	W-Beijing	1	Malaysia	1	Gly GGA 58 CGA Arg	Arg CGG 48 GGG Gly	Gly GGG 12 Gly GGA
43	W-Beijing	1	China	1	Gly GGA 58 CGA Arg	Arg CGG 48 GGG Gly	Gly GGG 12 Gly GGA
44	W-Beijing	1	Thailand	1	Gly GGA 58 CGA Arg	Arg CGG 48 GGG Gly	Gly GGG 12 Gly GGA
45	W-Beijing	1	Malaysia	1	Gly GGA 58 CGA Arg	Arg CGG 48 GGG Gly	Gly GGG 12 Gly GGA
91/102-6	W-Beijing	6	Vietnam	1	Gly GGA 58 CGA Arg	Arg CGG 48 GGG Gly	Gly GGG 12 Gly GGA
110/116/119/124-5/140-2	W-Beijing	8	the Netherlands	1	Gly GGA 58 CGA Arg	Arg CGG 48 GGG Gly	Gly GGG 12 Gly GGA
133	W-Beijing	1	South Africa	1	Gly GGA 58 CGA Arg	Arg CGG 48 GGG Gly	Gly GGG 12 Gly GGA
W4/10/126/129	W-Beijing	4	USA	1	Gly GGA 58 CGA Arg	Arg CGG 48 GGG Gly	Gly GGG 12 Gly GGA
W99	W-Beijing	1	Singapore	1	Gly GGA 58 CGA Arg	Arg CGG 48 GGG Gly	Gly GGG 12 Gly GGA
W147	W-Beijing	1	Russia	1	Gly GGA 58 CGA Arg	Arg CGG 48 GGG Gly	Gly GGG 12 Gly GGA
94	W-Beijing	1	Vietnam	1	wt	Arg CGG 48 GGG Gly	Arg CGC 37 Leu CTC
111	W-Beijing	1	South Korea	1	wt	Arg CGG 48 GGG Gly	Arg CGC 37 Leu CTC
115	W-Beijing	1	the Netherlands	1	wt	Arg CGG 48 GGG Gly	Arg CGC 37 Leu CTC
5107*(*HG1)	W-Beijing	1	USA	1	wt	Arg CGG 48 GGG Gly	Arg CGC 37 Leu CTC
114, 139	W-Beijing	1	the Netherlands	1	wt	Arg CGG 48 GGG Gly	wt
166(HD6)	W-Beijing	1	USA	1	wt	Arg CGG 48 GGG Gly	wt
165(001)	W-Beijing	1	USA	1	wt	wt	Arg CGC 37 Leu CTC
107(LB)	W-Beijing	1	USA	1	wt	wt	wt
113	W-Beijing	1	the Netherlands	1	wt	wt	wt
122(CI1)	W-Beijing	1	USA	1	wt	wt	wt
IK/KY/LB2/DV/DU2/HI	W-Beijing	6	Russia	1	wt	wt	wt
N16	W-Beijing	1	USA	1	wt	wt	wt
AM	W-Beijing	1	USA	1	wt	wt	wt

^a^wt, wild-type alleles (identical to H37Rv strain); nd, not determined.

**Table 2:** Characteristics of *Mycobacterium tuberculosis* complex strains originating from 35 different countries

Strains	Genotype	No. isolates	Country of isolation	Group	*mutT2*	mut T4	ogt
AU	Haarlem	1	USA	2	wt	wt	Thr ACC 15 Ser AGC
3,5,22,32,39,48,50,52-3,55	Haarlem	10	Argentina	2	wt	wt	Thr ACC 15 Ser AGC
8	Haarlem	1	Vietnam	2	wt	wt	Thr ACC 15 Ser AGC
13/28	Haarlem	2	Sri Lanka	2	wt	wt	Thr ACC 15 Ser AGC
51	Haarlem	1	the Netherlands	2	wt	wt	Thr ACC 15 Ser AGC
57/59	Haarlem	2	Czech republic	2	wt	wt	wt
84	Haarlem	1	Czech Republic	2	wt	wt	Thr ACC 15 Ser AGC
86/143/145	Haarlem	3	Bolivia	2	wt	wt	Thr ACC 15 Ser AGC
87	Haarlem	1	USA	2	wt	wt	Thr ACC 15 Ser AGC
99	Haarlem	1	Italy	2	wt	wt	Thr ACC 15 Ser AGC
123	Haarlem	1	Czech Republic	2	wt	wt	Thr ACC 15 Ser AGC
144/146-7	Haarlem	3	Bolivia	2	wt	wt	wt
Apr-35	Africa	2	Rwanda	2	wt	wt	wt
37	Africa	1	Uganda	2	wt	wt	wt
40/120	Africa	2	Burundi	2	wt	wt	wt
72	Africa	1	Central African Republic	2	wt	wt	wt
97	Africa	1	Uganda	2	wt	wt	wt
121	Africa	1	Central African Republic	2	wt	wt	wt
2	BCG	1	the Netherlands	1	wt	wt	wt
6/47/73/130	*M. bovis*	4	the Netherlands	1	wt	wt	wt
12	Other	1	Tunisia	3	wt	wt	wt
15/31	Other	2	Iran	2	wt	wt	wt
16	Other	1	Canada	2	wt	wt	wt
17	Other	1	Greenland	2	wt	wt	wt
18	Other	1	USA	2	wt	wt	wt
19/36/74	Other	2	India	1	wt	wt	wt
25/62	*M. microti*	2	UK	1	wt	wt	wt
26	Other	1	Zimbabwe	2	wt	wt	wt
27	Other	1	Ethiopia	2	wt	wt	wt
38/42	Other	2	Tahiti	2	wt	wt	wt
41/46	Other	2	Chile	2	wt	wt	wt
49	Other	1	Tanzania	1	wt	wt	wt
56	Other	1	Curacao	2	wt	wt	wt
64	Other	1	Honduras	2	wt	wt	wt
65/112	Other	2	the Netherlands	1	wt	wt	wt
71	BCG	1	Japan	1	wt	wt	wt
76/101/126	*M. bovis*	3	Argentina	1	wt	wt	wt
83	BCG	1	Russia	1	wt	wt	wt
89/95	Other	2	Spain	2	wt	wt	wt
96	Other	1	the Netherlands	3	wt	wt	wt
98	Other	1	Ecuador	2	wt	wt	wt
100	*M. africanum*	1	the Netherlands	1	wt	wt	wt
108	Other	1	China	2	wt	wt	wt
118	Other	1	Honduras	2	wt	wt	wt

^a^nd, not determined; wt, wild-type alleles (identical to H37Rv strain).

Primers were designed to amplify putative *mut* genes: *mutY* (5′-CCGGCGACGAATCGCTCGTT-3′, 5′-AGCTGGGACAGTCGTCGCGG-3′), *mutM* (5′-CTGGTTCGATGGTGATGACC-5′, 5′-GTGCGCTCGACCCACAG-3′), *mutT2* (5′-TCCGGATGATGATTTACCTCC-3′, 5′-TCCGCCGGGTCGGGGAC-3′), *mutT1* (5′-ATCGTCGGCGTGCCGTG-3′, 5′-GTCAGCGTCCTGCCCGG-3′), *mutT4* (5′-TCGAAGGTGGGCAAATCGTG-3′ 5′-TGGGGTTCGCTGGAAGTGG–3′), *ogt* (5′-CAGCGCTCGCTGGCGCC-3′, 5′-GACTCAGCCGCTCGCGA-3′), and *mut T3* (5′-GTCACGTCTGTTAGGACCTC-3′, 5′-CGCGCAACGGCTGCCGG-3′). Similar primers were designed to amplify the *rpoB* gene (5′-TACGGTCGGCGAGCTGATCC-3′, 5′- TACGGCGTTTCGATGAACC-3′).

DNA sequencing was performed directly on the amplified fragments by using the dideoxy chain-termination method with the Big Dye Terminator Cycle sequencing Kit (Perkin Elmer Applied Biosystems, Courtaboeuf, France) on a GeneAmp polymerase chain reaction (PCR) system 9600 (Perkin Elmer) and run on a DNA analysis system model 373 or 3100 (Applied Biosystems). Sequences of *mutY*, *mutT2*, *mutT4*, *rpoB, mutT1, mutT3,* and *ogt* of the *M. tuberculosis* strains H37Rv, CDC1551, and MT210 were obtained from published sequences or at the TIGR Web site (available from: URL: http://www.tigr.org/).

## Results

We searched for allele variation in putative genes coding for DNA repair enzymes: *mutT* (which hydrolyzes 8-oxo-deoxyguanosine triphosphate) (*28*), *ogt* (which removes methyl groups from O6-methylguanine in DNA) (*29*), *mutM* (formamidopyrimidine-DNA glycosylate) (*30*), and *mutY* (specific adenine glycosylate) (*31*) in 12 MDR *M. tuberculosis* strains. Subsequently, we genotyped for the observed single nucleotide polymorphism (SNP) variation in 124 strains, members of the *M. tuberculosis* complex, and in the three published sequences of *M. tuberculosis* strains. In total, the sampling comprises 55 W-Beijing genotype *M. tuberculosis* strains, including 11 ancestral W-Beijing isolates (unpub. data ), 84 *M. tuberculosis* strains of other genotypes, and 1 *M. bovis* strain.

**Figure 2:**
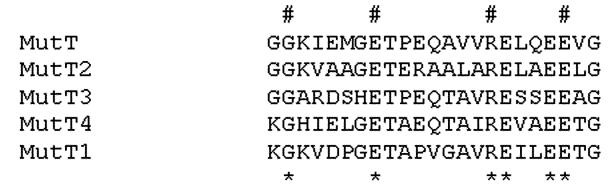
MutT proteins’ sequences alignment. *Mycobacterium tuberculosis* Rv2985(MutT1), Rv1160(MutT2), Rv0413(MutT3), and Rv3908(MutT4) were selected from the *M. tuberculosis* genome because of their annotation or after a BLAST analysis. These sequences were compared to *Escherichia coli* *mutT* by using alignments available from: http://www.biochem.uthscsa.edu/~barnes/mutt.html. The detected region of similarity is shown here. #, absolutely conserved residues; *, residues that are strongly conserved and that define the mutT or nudix motif.

Several putative *mut* genes were annotated as such in the released genome sequence of *M. tuberculosis*. A BLAST search using the *E. coli* *mutT* sequences as template identified, in addition to *mutT1*, *mutT2*, *mutT3*, the hypothetical ORF Rv3908, which we have designated as *mutT4*. The best matches with *E. coli* *mutT* gene were observed for *mutT2* and *mutT4*. Figure 2 depicts sequence alignment of the conserved region of the different genes of the *M. tuberculosis* genome showing similarity with *mutT* of *E. coli*. The search for sequences similar to *ogt,* *mutM,* and *mutY* identified a single ORF in each case. Primers were designed for PCR amplification of all the genes mentioned above.

We first determined the sequences of the different genes mentioned above in 12 MDR *M. tuberculosis* strains (ZA20, ZA65, ZA67, ZA68, ZA69, ZA11, ZA16, ZA12, ZA13, ZAA14, ZA17, and ZA19), including 5 W-Beijing strains (ZA20, ZA65, ZA67, ZA78, and ZA69). For the *mutY*, *mutM*, *mutT1*, and *mutT3* putative genes, PCR amplification was obtained in all strains tested, but sequence analysis did not indicate any nucleotide changes at these loci except for the same silent SNP in *mutT3* in strains with a Haarlem genotype. We confirmed these findings by sequencing *mutT1, mutT3*, *mutM,* and *mutY* in a collection of 26 MDR strains from North Africa. No variation was observed in *mutT1* or *mutM*. Only one strain had a major variation in *mutY*. All Haarlem strains carried one characteristic silent mutation in *mutT3* and one characteristic mutation in *ogt* (Ser 15 replaced by Thr). These defining SNPs were also observed for all Haarlem strains of this study. No other variations were observed in *mutT1, mutT3, mutM,* or *mutY*. However, comparative sequence analysis of H37Rv, CDC1551, and the five MDR–W-Beijing isolates indicated polymorphisms in *mutT2*, *mutT4,* and *ogt*. These mutations in *mutT4*, *mutT2,* and *ogt* were also found in the W-Beijing strain 210 (TIGR) but not in MDR strains other than those belonging to the W-Beijing genotype. We therefore decided to extend this investigation and look for mutations in these three genes in a collection of *M. tuberculosis* complex isolates, including well-defined branches of the W-Beijing phylogenetic lineage (Table 1 and 2).

In 43 of 55 strains with a W-Beijing genotype, either susceptible to bacteriostat or MDR, we found a mutation in *mutT4*. Codon 48 (CGG) of the annotated ORF had been changed to GGG, resulting in the amino acid substitution of Arg by Gly (Table 1 and 2). All 11 W-Beijing isolates known to be closely related to the ancestral W-Beijing strain (AM, HI, N16, DU2, DV, LB2, KY, IK, 122(C11), 113, and 107(LB)) were found to have the wild-type genotype as all other 84 isolates with a genotype other than W-Beijing.

Thirty-nine of 43 W-Beijing strains with the mutation in *mutT4* carried an additional mutation in *mutT2* and in *ogt*. The *mutT2* mutation constitutes a change in codon 58 (GGA to CGA), resulting in an amino acid substitution of Gly by Arg. The active site of the *E. coli* MutT enzyme comprises amino acids 53, 56, 57, and 98. Therefore, a mutation Gly to Arg at position 58 may have a important effect on enzyme activity and lead to a mutator phenotype.

All 39 W-Beijing isolates carrying the *mutT2* polymorphism at codon 58 also displayed a concurrent silent mutation in codon 12 (Gly GGG to GGA Gly) of the *ogt* gene. Of four possible codons encoding for glycine, GGG and GGA had the lowest relative synonymous codon usage (RSCU) in genes with high expression levels (0.20 and 0.17, respectively, compared to 1.32 and 2.31 for GGU and GGC). For genes with low expression levels, the RSCU values are 0.92, 0.37, 0.65, and 2.06 for GGG, GGA, GGU, and GGC, respectively (*32*).

**Figure 3:**
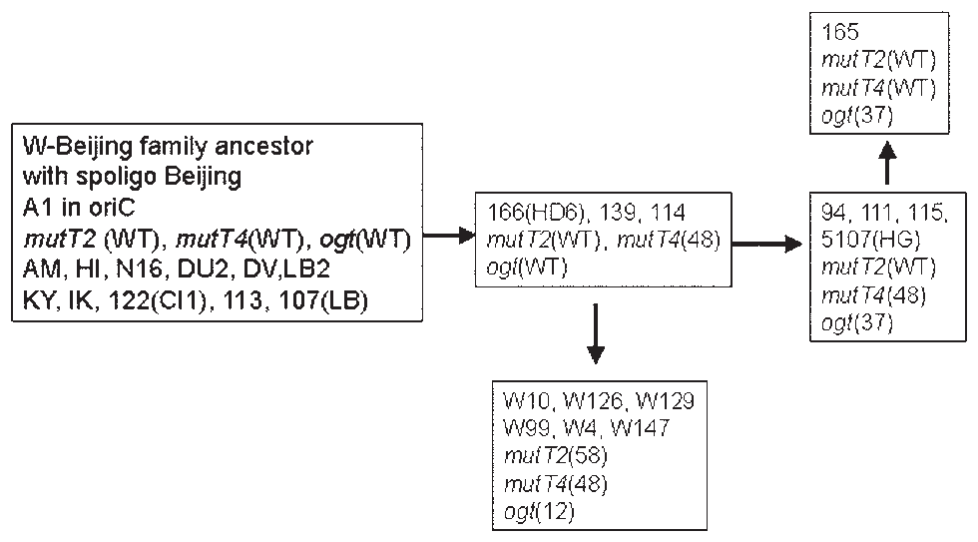
Schematic representation of a plausible pathway to explain the accumulation of mutations in *mut* genes.

The five W-Beijing isolates with a mutation in *mutT4* and a wild-type *mutT2* gene did not contain the *ogt* silent mutation on codon 12 either. Instead, they all shared a dinucleotide substitution in codon 37 (ACC to CTC) of *ogt*, resulting in amino acid substitution of Arg to Leu. These five W-Beijing isolates of 43 with the *mutT4* mutations, without the *mutT2* (codon 58) or the *ogt* (codon 12) mutations, differed molecularly from all other W-Beijing isolates in their spoligotype pattern and accompanying deletion flanking the DR locus. Four of five were isolated from Dutch patients in the Netherlands; the fifth originated from a patient in Vietnam. The Vietnamese isolate (no. 94) shared >95% IS*6110* pattern similarity with the Dutch isolate 115 when standard RFLP analysis was used. Overall, the five isolates were closely related to each other according to IS*6110* profiling (>90% similarity). Spacer 37 in the DR locus of Dutch isolates 114 and 139 was absent, while sample 115 was missing spacers 37 and 38, and 111 had a deletion of spacers 38 and 39 but not spacer 37, suggesting that these isolates may belong to a different sublineage. A tentative phylogeny of the W-Beijing strains analyzed in this study is proposed in Figure 3. Seven of nine MDR W-Beijing strains carried missense mutations in two *muT* genes (*mutT2* and *mutT4*), and two had a missense mutation in both *mutT4* and *ogt* (Table 1 and 2).

No mutations in *mutT4* or in *mutT2* were observed in any of the 84 *M. tuberculosis* complex strains, including 19 strains of PGG1, 54 strains of PGG2, and 2 strains of PGG3; the strains originated from 29 countries and were a genotype other than W-Beijing. A Thr15Ser mutation was observed in 24 of 29 strains of the Harlem family. No other change was observed in *ogt*.

Resistance to rifampicin in MDR strains was correlated with mutations in the *rpoB* gene. The three tested MDR W-Beijing strains isolated in Spain, with the mutations at the *mutT2* and *mutT4* loci, harbored a different mutation in the *rpoB* gene. These strains were isolated from patients who had emigrated from Eastern Europe to Spain (ZA67, ZA68, and ZA69). Analysis of the IS*6110* RFLP of the respective isolates showed a difference of a single band. These findings suggest that the three strains may be related. The acquisition of the three different mutations in the *rpoB* gene leading to rifampicin resistance must have occurred after the acquisition of mutations in the putative nucleotide repair enzyme genes *mutT4* and *mutT2*.

## Discussion

Our results show that *M. tuberculosis* strains of the W-Beijing genotype acquired missense mutations in DNA repair genes. These *M. tuberculosis* W-Beijing genotype strains are genetically highly conserved and widespread. DNA repair genes have been previously shown to be associated with mutator phenotypes in other microorganisms. The success of this group of strains may result in part from mutations in DNA repair enzymes, which might provided a true selective advantage for these bacteria to adapt and persist, including through the acquisition of resistance to anti-TB drugs. Mutations in the DNA repair genes might be the evolutionary answer of the TB bacillus to increase adaptation to hosts. This adaptation will lead to increasing trends in the TB epidemic in the coming decades. The World Health Organization considers MDR and resistance as a problem of local rather than of global importance (*1*). If the relative contribution of W-Beijing genotype strains to the current worldwide TB epidemic is increasing as suggested (*7*), this approach should be revised. In areas with an increasing problem with MDR-TB, such as Estonia and Russia, W-Beijing genotype strains are predominantly associated with MDR cases (*33*). In Germany, the relative proportion of W-Beijing strains among isolates from resistant cases has increased from 12% in 1995 to 35% in 2000 (unpub. data). The latter observation may indicate an increasing influence of W-Beijing strains on the worldwide TB epidemic.

We identify polymorphisms in *M. tuberculosis* in genes that might result in a mutator phenotype and therefore a plausibly better adaptation of the bacilli to a hostile environment (*34*). Forty-three of 55 W-Beijing isolates analyzed were found to have a unique mutation on the ORF Rv3908. This ORF contains a MutT domain and is denoted here as *mutT4*. Thirty-nine of 43 W-Beijing strains carried an additional and identical mutation in a second putative gene of the *mutT* family, *mutT2*, and an identical silent mutation in *ogt*.

The W-Beijing phylogenetic lineage probably acquired the mutation on codon 48 of the *mutT4* only once and before other mutations associated with the mutator genes we describe. This mutation clearly distinguishes ancestral W-Beijing isolates from contemporary W-Beijing strains. The 11 W-Beijing isolates that did not have the characteristic *mutT4* mutation on codon 48, consist of a collection of isolates known to be ancestral within this phylogenetic lineage, as determined by various other molecular techniques (unpub. data).

Nine of W-Beijing strains with the wild-type *mutT2* gene had a characteristic mutation on codon 37 of the *ogt* gene, which suggests that these isolates constitute a branch of the W-Beijing family that diverged after the acquisition of the *mutT4* mutation but before the development of the nucleotide substitution on *mutT2*. One strain carries the mutation 37 in *ogt* but no mutation in *mutT4*, a reversion that might have occurred after a transient mutator phenotype.

A mutation in *mutT2* was always associated with a mutation in *muT4*. A first mutation may have occurred in *mutT4* and thereafter a second mutation either in *mutT2* or *ogt* was acquired. As observed for other bacterial populations, mutator phenotypes may be transient in many cases to limit deleterious effects (*35*). Identifying these mutations may aid in the identification of *mut* genes in *M. tuberculosis*. These mutations associated with mutator genes provide a reliable tool for the identification of W-Beijing isolates and thus a useful marker for strains endowed with capacity to yield epidemics. The biologic consequences of these mutations and function of these DNA repair genes are currently been investigated in the laboratory.

Nine MDR strains with a W-Beijing genotype were among strains carrying two missense mutations in putative mutator genes. Phylogenetically unrelated *M. tuberculosis* MDR isolates had no mutations within the DNA repair genes investigated in this study. Our data support the idea that *M. tuberculosis* strains of the W-Beijing genotype may have adapted to hostile environments, including exposure to anti-TB drugs, because of a succession of alterations of DNA repair enzymes. Other genes involved in other DNA repair mechanisms or in the fidelity of DNA replication may also be involved and remain to be investigated.

The acquisition of mutator alleles was described as an adaptive response of bacteria to a succession of different environments (*18*,*35*,*36*). After infecting a host, *M. tuberculosis* has to adapt to different environments such as alveolar macrophages and dendritic cells and subsequently to granuloma containing inactivated macrophages or to activated macrophages after induction of the acquired immune responses. In addition, the bacilli have to adapt to the caseous media with low oxygen concentration in the center of tubercles and to different types of tissues during dissemination of the disease. Such variable growth conditions might select for mutations in *M. tuberculosis* strains, as described in other bacterial populations exposed to different environmental challenges. Mutations and selection might occur with an increased frequency caused by the toxic radicals produced in phagocytic cells.

However, a mutator phenotype is often transient. Otherwise a continual accumulation of mutations would lead to deleterious effects and loss of fitness. No difference in the frequency of spontaneous mutations, resulting in a rifampin resistance phenotype, was observed for W-Beijing strains (*37*). We suggest that a transient mutator phenotype allowed a better adaptation of W-Beijing strains. Subsequent compensatory mutations occurred to reverse the mutator phenotype. An alternative hypothesis would be the existence of a higher mutation rate in specific conditions (i.e., in mutagenic radicals inside phagocytes). The accumulation of mutations leading to antibiotic resistance in W-Beijing strains may be a consequence of the appearance of strains with a better adaptation to the hosts. MDR strains would be easily selected when patients with strains that have adapted better received inadequate anti-TB regimens.
